# Efficacy, safety, and tolerability of lebrikizumab in adolescent patients with uncontrolled asthma (ACOUSTICS)

**DOI:** 10.1002/clt2.12176

**Published:** 2022-07-14

**Authors:** Stanley J. Szefler, Graham Roberts, Adalberto S. Rubin, Stefan Zielen, Piotr Kuna, Oral Alpan, Judith Anzures‐Cabrera, Qiang Chen, Cécile T. J. Holweg, Janusz Kaminski, Wendy S. Putnam, John G. Matthews, Nikhil Kamath

**Affiliations:** ^1^ Department of Pediatrics Children's Hospital Colorado and the University of Colorado School of Medicine Anschutz Medical Campus Aurora Colorado USA; ^2^ University of Southampton School of Medicine and Southampton Biomedical Research Centre University Hospital Southampton NHS Foundation Trust Southampton UK; ^3^ David Hide Asthma and Allergy Research Centre Isle of Wight UK; ^4^ Federal University of Health Sciences of Porto Alegre and Santa Casa de Misericórdia Hospital Porto Alegre Brazil; ^5^ Goethe‐Universität Klinik für Kinder‐ und Jugendmedizin Frankfurt Germany; ^6^ Medical University of Łódź Lodz Poland; ^7^ O&O Alpan LLC Fairfax Virginia USA; ^8^ Roche Products Ltd Welwyn Garden City UK; ^9^ Roche (China) Holding Ltd Shanghai China; ^10^ Genentech, Inc. South San Francisco California USA; ^11^ Present address: Abbvie, IL USA; ^12^ Present address: MSD London UK; ^13^ Present address: Ultragenyx Pharmaceutical, Novato CA USA; ^14^ Present address: 23andMe, South San Francisco CA USA

**Keywords:** adolescents, IL‐13, lebrikizumab, uncontrolled asthma

## Abstract

**Background:**

Lebrikizumab is a monoclonal antibody that modulates activity of interleukin‐13. The Phase 3 ACOUSTICS study assessed lebrikizumab efficacy and safety in adolescents with uncontrolled asthma despite standard‐of‐care treatment.

**Methods:**

Adolescents (aged 12–17 years) with uncontrolled asthma, prebronchodilator forced expiratory volume in 1 s 40%–90% predicted, and stable background therapy were randomised 1:1:1 to receive lebrikizumab 125 or 37.5 mg or placebo subcutaneously once every 4 weeks. Primary efficacy endpoint was asthma exacerbation rate over 52 weeks.

**Results:**

Between August 2013 and July 2016, 579 patients were screened and 346 were randomised; 224 (65%) completed the study with 52 weeks of treatment. Lebrikizumab 125 mg (*n* = 116) reduced the exacerbation rate at 52 weeks versus placebo (*n* = 117; adjusted rate ratio [RR] 0.49 [95% CI 0.28–0.83]; 51% rate reduction). Lebrikizumab 37.5 mg (*n* = 113) was less effective at reducing exacerbations (RR 0.60 [95% CI 0.35–1.03]; 40% rate reduction). In patients with blood eosinophil counts ≥300 cells/μl, both lebrikizumab doses reduced exacerbations (125 mg: RR 0.44 [95% CI 0.21–0.89]; 37.5 mg: 0.42 [95% CI 0.19–0.93]). Treatment‐emergent adverse events, serious adverse events, and adverse events leading to study discontinuation occurred in 155 (68%), 7 (3%), and 5 (2%) of 229 patients who received lebrikizumab (both 125 and 37.5 mg doses) and in 72 (62%), 4 (3%), and 1 (1%) of 117 who received placebo, respectively. No deaths occurred.

**Conclusion:**

Lebrikizumab 125 mg reduced asthma exacerbation rates in adolescents with uncontrolled asthma. However, the study was prematurely terminated (sponsor's decision) potentially limiting interpretation of results.

**Clinical trial registration:**

NCT01875003 (www.ClinicalTrials.gov).

## 1 INTRODUCTION

Asthma is a heterogeneous disease characterised by chronic inflammation of the airways and is associated with airway hyperresponsiveness that leads to recurrent episodes of wheezing, breathlessness, chest tightness, and coughing, particularly at night or in the early morning.^1^ Asthma is a global health problem that affects approximately 339 million individuals worldwide.^2^ Other published studies reporting on the burden of asthma morbidity in adolescent patients (aged 12–17 years) have indicated that approximately 55%–65% of adolescent patients with severe or difficult‐to‐treat asthma receive at least three long‐term asthma control medications concomitantly.^3^, ^4^, ^5^ One study that included 364 adolescent patients with asthma found that, despite treatment, 44% of these patients required a corticosteroid burst and 19% received treatment in an emergency department ≤3 months prior to study baseline.^4^ Therefore, patients who have uncontrolled disease despite treatment with high‐dose inhaled corticosteroids (ICS) and a second controller represent a population with high unmet medical need. Even with guideline‐based asthma therapy,^1^ ≤50% of patients have treatment‐refractory or well‐controlled asthma.^6^ The variable response to standardised therapy may be due to the heterogeneity of asthma.^7^, ^8^, ^9^, ^10^

Interleukin (IL)‐13 is a pleiotropic type 2 cell cytokine thought to be a key contributor to the pathogenesis of asthma, affecting mucus production, bronchial fibrosis, immunoglobulin E (IgE) production, and smooth‐muscle hyperplasia as well as inflammatory‐cell recruitment and activation.^11^, ^12^, ^13^, ^14^ Lebrikizumab is a monoclonal antibody that binds specifically to IL‐13 with very high affinity, prevents the formation of the IL‐4Rα/IL‐13Rα1 heterodimerisation and downstream signalling, and does not interfere with endogenous regulation of IL‐13 activity via IL‐13Rα2 binding.^15^, ^16^, ^17^ Results from Phase 2 studies showed that treatment with lebrikizumab was associated with improvements in lung function and the rate of asthma exacerbations in patients with uncontrolled asthma, particularly in patients with high periostin levels.^15^, ^18^ Replicate Phase 3, randomised, controlled trials (LAVOLTA I/II) were conducted to further assess the efficacy and safety of lebrikizumab in adult patients with uncontrolled asthma despite treatment with standard‐of‐care medications. To help identify those patients hypothesised to most likely benefit from lebrikizumab treatment, they were classified by type 2 biomarker status. However, lebrikizumab did not consistently show statistically significant reductions in asthma exacerbations in biomarker‐high adult patients (periostin ≥50 ng/ml, blood eosinophil count ≥300 cells/μl, or both); the primary endpoint was met in LAVOLTA I but not in LAVOLTA II. Although a non‐significant numerical trend favouring lebrikizumab was observed, the study sponsor (Roche/Genentech) decided to terminate the lebrikizumab program.^19^

ACOUSTICS was a Phase 3 study to investigate the efficacy and safety of lebrikizumab in adolescent patients with uncontrolled asthma despite treatment with standard‐of‐care medications. The study was prematurely closed by the study sponsor as of July 2016; dosing and further enrolment in ACOUSTICS were closed and all patients were transitioned to safety follow‐up. Here, we report the findings from 346 patients (of 375 planned) enrolled in the study.

## 2 METHODS

### 2.1 Study design and participants

ACOUSTICS was a randomised, multicentre, double‐blind, placebo‐controlled, parallel‐group study that evaluated the efficacy, safety, and tolerability of lebrikizumab in adolescents with asthma whose disease remained uncontrolled despite daily treatment with ICS and at least one additional controller medication (e.g., long‐acting *β*‐agonists [LABAs], leukotriene receptor antagonists [LTRAs], long‐acting muscarinic antagonists [LAMAs], or theophylline). The protocol was amended once to mitigate recruitment challenges associated with the patient population and study design (see Appendix in Supporting Information S1 for complete study protocol).

**FIGURE 1:**
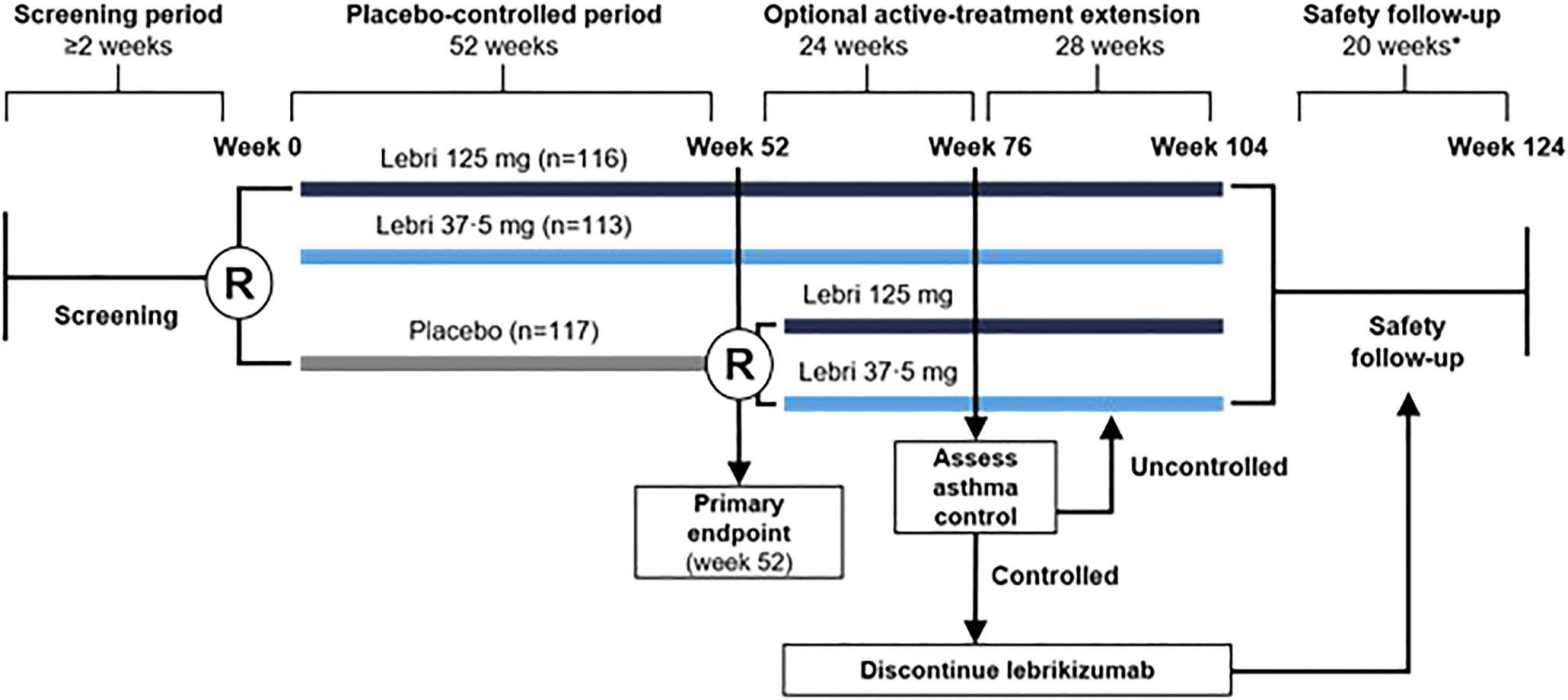
Study design schematic. Lebri, lebrikizumab; *R*, masked randomisation. *After the last dose of study drug during the optional active‐treatment extension (or during the placebo‐controlled period for patients who opt not to participate in the active‐treatment extension), all patients were to be followed‐up for safety for 24 weeks. This includes the 4 weeks after the final dose during the placebo‐controlled period or the active‐treatment extension and a 20‐week safety follow‐up period

The study consisted of a 2‐week screening period (Visits 1–3), a 52‐week placebo‐controlled period, an active‐treatment extension, and a safety follow‐up period (Figure 1). All patients participated in the placebo‐controlled period until Week 52, after which they could opt to participate in the active‐treatment extension. Patients who decided not to participate in the active‐treatment extension transitioned to the safety follow‐up period. At the termination of the study by the sponsor, all patients who were in the active‐treatment extension were transitioned to the safety follow‐up period or discontinued per patient's choice. Efficacy analyses of the 52‐week placebo‐controlled period are reported here.

Eligible patients were aged 12–17 years with uncontrolled asthma diagnosed ≥12 months previously, with pre‐bronchodilator forced expiratory volume in 1 s (FEV_1_) 40%–90% predicted and bronchodilator response of ≥12% and had been receiving background therapy with ICS (500–2000 μg/day fluticasone propionate dry‐powder inhaler or equivalent) for ≥6 months. Patients were also required to be receiving at least one additional controller medication (LABAs, LTRAs, LAMAs, or theophylline) for ≥6 months prior to Visit 1 (screening), with no changes within 4 weeks prior to Visit 1 and no anticipated changes throughout the study (except for theophylline dose, which could be adjusted on the basis of theophylline levels). During the screening period, patients were also assessed for adherence to their current asthma‐controller therapy, for their ability to use the equipment necessary for all visits throughout the study, and for complete data in a daily electronic diary (e‐Diary) reporting the degree of asthma control provided by their standard‐of‐care asthma medications. Adherence was defined as affirmative responses from patients that they had taken their asthma‐controller therapy on ≥70% of days during the screening period as recorded in their e‐Diary. Uncontrolled asthma was defined as a Five‐Item Asthma Control Questionnaire (ACQ‐5) score of ≥1.5 and at least one of the following asthma symptoms that were not controlled during the screening period: symptoms for >2 days/week, night time awakenings at least once per week, use of a short‐acting *β*‐agonist as rescue medication for >2 days/week, or interference with normal daily activities. Exclusion criteria included a history of a severe allergic or anaphylactic reaction to a biologic agent or known hypersensitivity to any component of the lebrikizumab injection, maintenance oral corticosteroid therapy in the last 3 months, systemic corticosteroid therapy in the last 4 months, clinically significant lung disease other than asthma, infection requiring hospital admission or treatment with intravenous or intramuscular antibiotics in the last 4 weeks, any active infection that required treatment with oral antibiotics in the last 2 weeks, upper or lower respiratory tract infection in the last 4 weeks, active parasitic infection or *Listeria monocytogenes* infection in the last 6 months, and history of active tuberculosis requiring treatment.

This study was conducted in accordance with the Declaration of Helsinki and the International Council for Harmonisation Guideline for Good Clinical Practice. Independent ethics committee approval was obtained at all participating centres, and all patients provided written informed assent, where appropriate, with consent from legal guardians. An independent data monitoring committee reviewed safety data at regular intervals throughout the trial.

### 2.2 Dose selection, randomisation, and masking

Two dose levels were investigated in the study: the 125‐mg dose was expected to demonstrate clinical efficacy while the 37.5‐mg dose was selected to ensure minimal overlap in the range of serum exposures between the two doses. Following completion of the screening period and after all patient eligibility requirements were confirmed, patients were randomised (at Visit 3) 1:1:1 to receive lebrikizumab 125 mg, lebrikizumab 37.5 mg, or placebo every 4 weeks during the placebo‐controlled period. Treatment was initiated on the same day as randomisation (Visit 3, Day 1).

Randomisation was stratified by history of asthma exacerbations within the last 12 months (0, 1–2, or ≥3 events), baseline asthma medications (ICS total daily dose ≥1000 μg of fluticasone propionate dry‐powder inhaler or equivalent plus LABA [yes or no]), age group (12–14 or 15–17 years), and country. A dynamic randomisation method was used to obtain an approximate 1:1:1 ratio among the three treatment groups and within each stratum. Patients who opted to transition to the active‐treatment extension and who had been assigned to placebo during the placebo‐controlled period were re‐randomised 1:1 to lebrikizumab 125 mg or 37.5 mg every 4 weeks. For these patients, randomisation into the active‐treatment extension was performed using a block design stratified by age group (12–14 or 15–17 years).

### 2.3 Procedures

Lebrikizumab 125 mg and 37.5 mg were given subcutaneously every 4 weeks during the placebo‐controlled period and the optional active‐treatment extension period. Assessments during the study included recording of exacerbation events, healthcare use, spirometry, patient‐reported outcome questionnaires, and adverse events. Pharmacokinetics, pharmacodynamics biomarkers, and antidrug antibodies were measured at Weeks 4, 12, 24, 36, and 52 (see Appendix in Supporting Information S1 for further details). Patients were provided with a handheld peak flow metre and an e‐Diary, which were used for once‐daily peak expiratory flow measurements and to record use of asthma rescue and controller medication during the study.

### 2.4 Outcomes

The primary study endpoint was the asthma exacerbation rate over 52 weeks. An asthma exacerbation was defined as new or worsened asthma symptoms that led to treatment with systemic corticosteroids or to hospital admission. Treatment with systemic corticosteroids was defined as oral, intravenous, or intramuscular corticosteroid therapy for ≥3 days or at least one dose of intravenous or intramuscular corticosteroids administered during an emergency department visit.

Secondary endpoints included relative change in prebronchodilator FEV_1_ from baseline to Week 52; time to first asthma exacerbation during the 52‐week placebo‐controlled period; change in fractional exhaled nitric oxide (FeNO) from baseline to Week 52; change in asthma‐specific health‐related quality of life from baseline to Week 52, as assessed by the overall score of the Asthma Quality of Life Questionnaire for 12 years and older (AQLQ +12); change in asthma rescue medication use from baseline to Week 52; and rate of urgent asthma‐related healthcare use (i.e., hospitalisations, emergency department visits, and acute care visits) during the 52‐week placebo‐controlled period.

Safety outcomes included the frequency and severity of adverse events during the 52‐week placebo‐controlled period and the safety follow‐up period and incidence of antidrug antibodies against lebrikizumab.

An exploratory endpoint included changes in ACQ‐5 score during the placebo‐controlled period; because the study was closed prematurely, the remaining exploratory endpoints were not analysed (see Appendix in Supporting Information S1 for study protocol).

The pharmacokinetic objective of the study was to evaluate serum lebrikizumab concentrations over the 52‐week placebo‐controlled period.

Absolute change from baseline in blood eosinophil count, serum C‐C motif chemokine 13 (CCL13), and serum total IgE were measured throughout the 52‐week placebo‐controlled period as biomarkers of IL‐13 activity.

### 2.5 Statistical analysis

The primary and secondary efficacy analyses were assessed in the intention‐to‐treat population, in which all randomised patients were grouped by the treatment assigned at randomisation. Per the planned study protocol, hypothesis‐testing for the efficacy endpoints was to be performed between each lebrikizumab dose level and the placebo group. To manage the overall type I error, comparison of efficacy in the lebrikizumab 37.5‐mg group with that in the placebo group was to be gated on the success of the primary endpoint test in the lebrikizumab 125‐mg group compared with the placebo group. Because the study was closed early with no intention of using the results for decision‐making purposes, all findings are presented descriptively as point estimates with 95% CIs.

It was estimated that a total of 375 adolescents would be needed to achieve approximately 80% power to detect a 50% reduction in the asthma exacerbation rates with a given lebrikizumab dose level compared with placebo.

The primary efficacy endpoint was the asthma exacerbation rate during the 52‐week placebo‐controlled period. For each treatment group, the unadjusted asthma exacerbation rate was estimated by the total number of exacerbations observed during the placebo‐controlled period divided by the total patient‐weeks at risk in the group. For each patient, the period at risk was extended from the day of randomisation to the day of early discontinuation from the placebo‐controlled period.

The asthma exacerbation rates were compared between each active‐treatment group and the placebo group by use of a Poisson regression model with overdispersion. Analyses were based on observed exacerbations over the period at risk. A patient's time at risk, as defined above, was used as an offset term in the model. The Poisson regression model was stratified by age group, history of asthma exacerbation in the last 12 months, and baseline asthma medication. Adjusted asthma exacerbation rates were estimated using the Poisson regression model for each treatment group. The relative reduction in the exacerbation rate in each lebrikizumab group compared with the placebo group was estimated by the exponentiated treatment coefficient, and the treatment coefficient from the Poisson regression model.

For analyses of secondary endpoints, a Cox proportional hazards regression model was used to estimate the hazard ratio (HR), comparing each lebrikizumab treatment group with the placebo group. Patients who did not experience a protocol‐defined asthma exacerbation were censored at the date of the Week 52 visit or at the date of early discontinuation from the study. The mean changes from baseline in prebronchodilator FEV_1_, FeNO, AQLQ +12 scores, and asthma rescue medication use were analysed using a mixed‐effects repeated‐measures model. Analysis of the rates of urgent asthma‐related healthcare use was performed using Poisson regression with overdispersion similar to that used for protocol‐defined asthma exacerbations. Efficacy analyses in this study were also stratified by baseline blood eosinophil levels (≥300 and <300 cells/μl).

Safety analyses were based on all patients who received at least one dose of study drug, with patients grouped by the actual treatment received. Safety was assessed through summary of adverse events, laboratory test results, and incidence of antibodies against lebrikizumab.

ACOUSTICS was registered with ClinicalTrials.gov, number NCT01875003.

### 2.6 Role of the funding source

The sponsor of the study contributed to study design, data collection, data analysis, data interpretation, and writing of the report. All authors had full access to all the data in the study, and the corresponding author had final responsibility for the decision to submit for publication.

## 3 RESULTS

**FIGURE 2:**
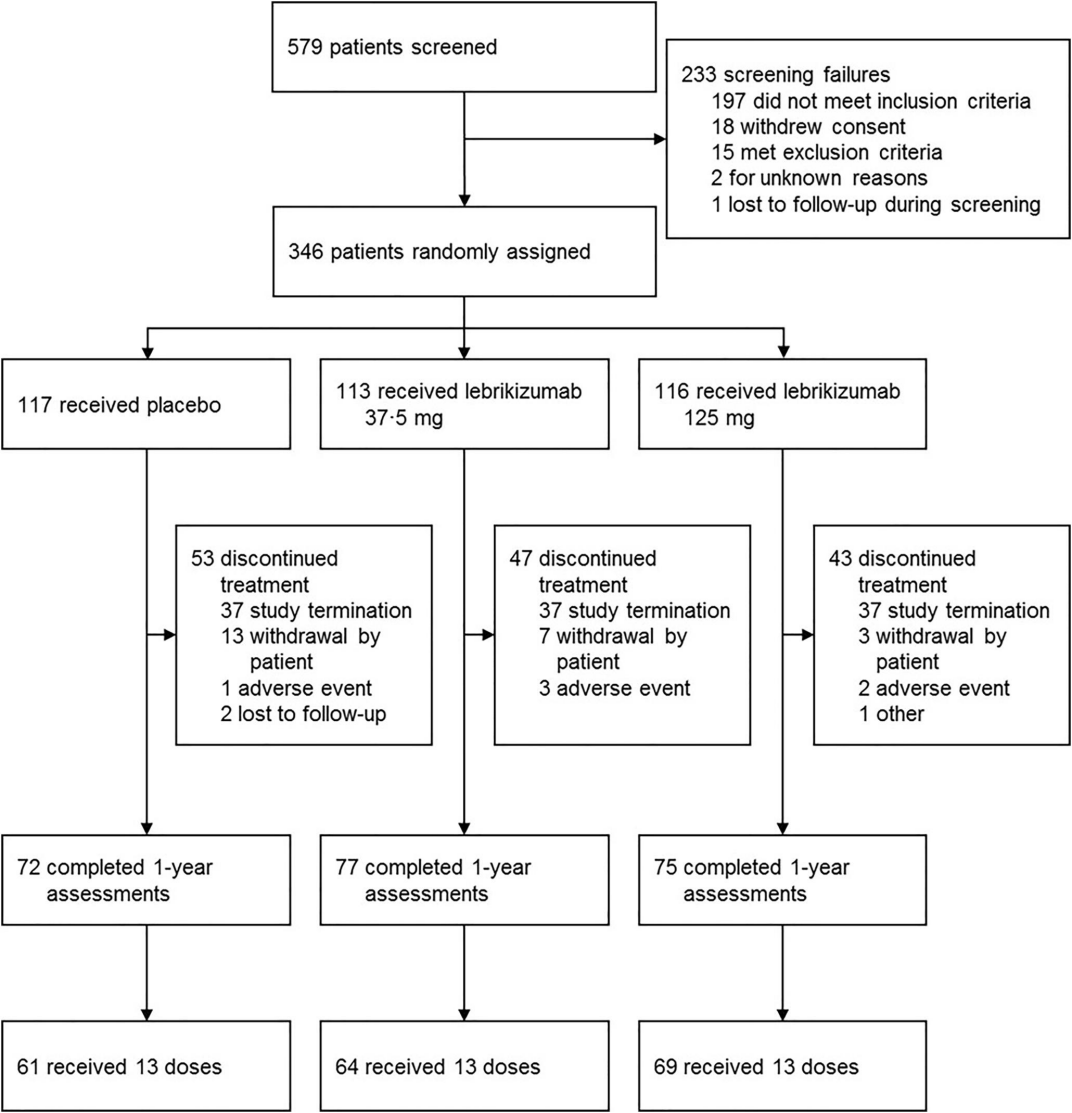
Trial profile

Beginning August 2013, 579 patients were screened at 78 sites in 20 countries (Appendix in Supporting Information S1). Of these 579 patients, 346 were randomised prior to the premature closure of enrolment on July 4, 2016. All patients who were randomised received at least one dose of study drug (intention‐to‐treat population). When study dosing was closed, 224 of 346 patients (65%) completed the 52‐week placebo‐controlled period, and 133 of 229 patients treated with lebrikizumab (58%) received the full 13 doses of study drug (Figure 2). A total of 146 patients discontinued dosing prematurely within the placebo‐controlled period and directly entered the safety follow‐up period, without entering the optional active‐treatment extension phase (Figures 1 and 2). The last safety follow‐up patient visit occurred on December 28, 2016.

Baseline characteristics were similar across treatment groups, with a few exceptions (Table 1). The proportion of patients with eosinophil counts ≥300 cells/μl was different across treatment groups, with the highest proportion in the placebo group (56%) and the lowest in the lebrikizumab 37.5‐mg group (44%).

**TABLE 1:** Patient baseline characteristics in the intention‐to‐treat population

	Placebo (*n* = 117)	Lebrikizumab 37.5 mg (*n* = 113)	Lebrikizumab 125 mg (*n* = 116)	All patients (*N* = 346)
Age, years	14.1 (1.7)	14.2 (1.5)	14.2 (1.6)	14.2 (1.6)
Sex				
Male	68 (58%)	70 (62%)	57 (49%)	195 (56%)
Female	49 (42%)	43 (38%)	59 (51%)	151 (44%)
Age group, years				
12–14	71 (61%)	64 (57%)	67 (58%)	202 (58%)
15–17	46 (39%)	49 (43%)	49 (42%)	144 (42%)
White race	84 (72%)	75 (66%)	74 (64%)	223 (67%)
Body mass index, kg/m^2^	22.1 (4.0)	22.4 (4.6)	22.8 (5.0)	22.4 (4.6)
Median duration of asthma (range), years	9 (1–17)	11 (1–17)	11 (1–17)	11 (1–17)
Mean ICS (fluticasone propionate DPI or equivalent), μg/day	699.8 (397.8)	751.8 (621.3)	672.0 (416.9)	707.4 (487.4)
Baseline LABA	107 (91)	101 (89)	106 (91)	314 (91)
Prebronchodilator FEV_1_				
Absolute, L	2.220 (0.542)	2.393 (0.494)	2.266 (0.482)	2.292 (0.510)
% Predicted	69.8 (12.5)	73.3 (9.9)	70.9 (10.7)	71.3 (11.2)
ACQ‐5 score (0–6)	2.76 (0.83)	2.57 (0.84)	2.69 (0.90)	2.67 (0.86)
AQLQ +12 score (1–7)	4.42 (1.09)	4.65 (1.15)	4.22 (1.26)	4.42 (1.18)
Median IL‐13, pg/ml	1.4 (0.8–2.3)^a^	1.2 (0.8–1.8)^†^	1.3 (0.8–2.2)^c^	1.3 (0.8–2.1)
Median CCL13, pg/ml	149 (119–204)	169 (123–231)^§^	154 (110–187)	156 (118–204)
Median IgE, IU/ml	358 (156–984)	361 (126–1027)^§^	368 (170–921)^e^	361 (154–984)
Median FeNO, ppb^f^	30 (14–59)^g^	37 (18–62)^c^	33 (16–58)^e^	33 (16–59)
Median blood eosinophil count, cells/μl^f^	330 (190–530)	280 (140–420)	295 (180–515)	295 (160–490)
Elevated eosinophil count (≥300 cells/μl)^f^	65 (56%)	50 (44%)	58 (50%)	173 (50%)
Patients with ≥1 exacerbation in previous 12 months	76 (65%)	70 (63%)^e^	83 (72%)	229 (66%)^e^

*Note*: Data are *n* (%), mean (SD), or median (IQR), unless otherwise stated.

Abbreviations: ACQ‐5, Five‐Item Asthma Control Questionnaire; AQLQ +12, Asthma Quality of Life Questionnaire for 12 years and older; CCL13, C‐C motif chemokine 13; DPI, dry‐powder inhaler; feno, fractional exhaled nitric oxide; FEV_1_, forced expiratory volume in 1 s; ICS, inhaled corticosteroid; IgE, immunoglobulin E; IL, interleukin; LABA, long‐acting *β*‐agonist; ppb, parts per billion.

^a^ Three patients missing.

^b^ Eleven patients missing.

^c^ Two patients missing.

^d^ Four patients missing.

^e^ One patient missing.

^f^ Measured at screening.

^g^ Six patients missing.

**FIGURE 3:**
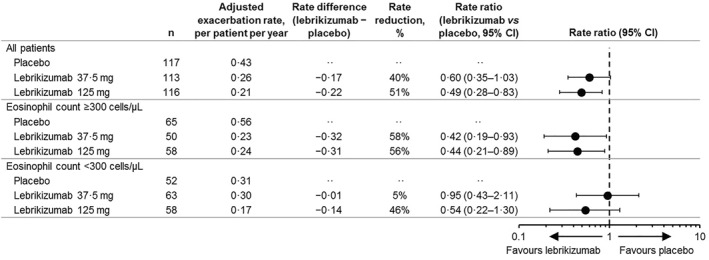
Adjusted rate of asthma exacerbations over 52 weeks in all patients and by eosinophil group

A total of 113 exacerbations were reported during the 52‐week placebo‐controlled period, including 51 in patients receiving placebo (98.4 patient‐years of follow‐up) and 31 each in patients treated with lebrikizumab 125 and 37.5 mg (105.1 and 100.8 patient‐years at risk, respectively). Overall, treatment with lebrikizumab reduced the asthma exacerbation rate compared with placebo over 52 weeks (Figure 3). Compared with placebo, the adjusted rate ratio [RR] in the lebrikizumab 125‐mg group was 0.49 (95% CI 0.28 to 0.83), corresponding to a 51% reduction in asthma exacerbation rates. In the lebrikizumab 37.5‐mg group, the RR was 0.60 (95% CI 0.35–1.03), corresponding to a 40% reduction in asthma exacerbation rates.

When patients were stratified by blood eosinophil counts (≥300 vs. <300 cells/μl) both lebrikizumab doses demonstrated a greater exacerbation rate reduction in higher versus lower eosinophil counts (Figure 3). Among patients with high blood eosinophil counts, the adjusted RRs were 0.44 (95% CI 0.21–0.89) in the lebrikizumab 125‐mg group and 0.42 (95% CI 0.19–0.93) in the lebrikizumab 37.5‐mg group compared with placebo (corresponding to asthma exacerbation rate reductions of 56% and 58%, respectively). Among patients with low blood eosinophil counts, the adjusted RRs were 0.54 (95% CI 0.22–1.30) in the lebrikizumab 125‐mg group and 0.95 (95% CI 0.43–2.11) in the lebrikizumab 37.5‐mg group compared with the placebo group.

**FIGURE 4:**
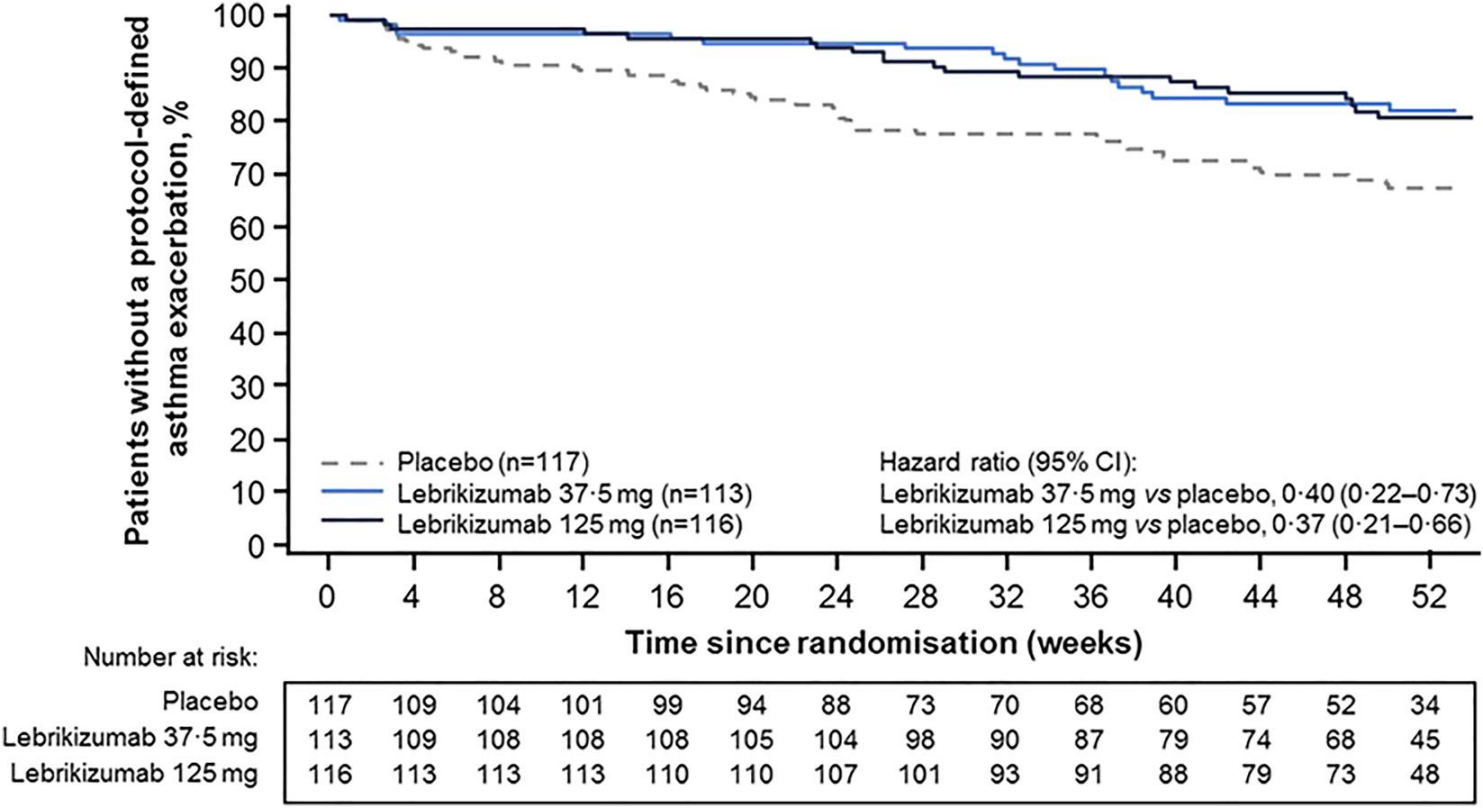
Time to first asthma exacerbation

An overview of the secondary and exploratory efficacy endpoints is presented in Table 2. A trend towards increased prebronchodilator FEV_1_ was observed in patients treated with lebrikizumab 37.5 mg; the placebo‐corrected mean change from baseline to Week 52 was 198 ml (95% CI 53–342 ml) in the lebrikizumab 37.5‐mg group and 53 ml (95% CI −92 to 198 ml) in the lebrikizumab 125‐mg group (see Appendix in Supporting Information S1 for mean change in FEV_1_ over time). Patients treated with lebrikizumab had an increased time to first asthma exacerbation compared with those receiving placebo (HR 0.37 [95% CI 0.21–0.66] in the lebrikizumab 125‐mg group and HR 0.40 [95% CI 0.22–0.73] in the lebrikizumab 37.5‐mg group) (Table 2 and Figure 4). Patients receiving lebrikizumab also had a reduction from baseline in FeNO; the placebo‐corrected mean change from baseline to Week 52 was −30.3 parts per billion (ppb; 95% CI −37.9 to −22.8 ppb) in the lebrikizumab 125‐mg group and −22.0 ppb (95% CI −29.4 to −14.6 ppb) in the lebrikizumab 37.5‐mg group. Although the overall number of events of urgent asthma‐related healthcare use was low over 52 weeks (Table 2), a decreased rate of events was observed among patients treated with either lebrikizumab dose compared with patients treated with placebo (RR 0.27 [95% CI 0.10 to 0.72] with lebrikizumab 125 mg and RR 0.40 [95% CI 0.16 to 1.00] with lebrikizumab 37.5 mg). No evidence of a change in rescue medication use or quality of life was observed as assessed by the AQLQ +12 with either lebrikizumab dose. (See Appendix in Supporting Information S1 for ACQ‐5, pharmacodynamics, pharmacokinetics, and antidrug antibody assessment results.)

**TABLE 2:** Secondary and exploratory efficacy estimates at 52 weeks (ITT population)

	Placebo (*n* = 117)	Lebrikizumab 37.5 mg (*n* = 113)	Lebrikizumab 125 mg (*n* = 116)
Change from baseline in prebronchodilator FEV_1_, ml^a^			
Adjusted mean (SE)	370 (54)	568 (53)	423 (54)
Difference in means versus placebo (95% CI)	..	198 (53–342)	53 (−92–198)
Time to first exacerbation			
Patients with event, *n* (%)	33 (28%)	18 (16%)	20 (17%)
Hazard ratio versus placebo (95% CI)	..	0.40 (0.22 to 0.73)	0.37 (0.21 to 0.66)
Change from baseline in prebronchodilator FeNO, ppb^a^			
Adjusted mean (SE)	3.9 (2.8)	−18.0 (2.7)	−26.4 (2.8)
Difference in means versus placebo (95% CI)	..	−22.0 (−29.4 to −14.6)	−30.3 (−37.9 to −22.8)
Change from baseline in ACQ‐5 score			
Adjusted mean (SE)	−1.04 (0.11)	−1.38 (0.11)	−1.34 (0.11)
Difference in means versus placebo (95% CI)	..	−0.34 (−0.63 to −0.05)	−0.30 (−0.59 to 0.00)
Change from baseline in AQLQ +12 score^b^			
Adjusted mean (SE)	1.05 (0.11)	1.17 (0.11)	1.21 (0.11)
Difference in means versus placebo (95% CI)	..	0.12 (−0.18 to 0.41)	0.15 (−0.14 to 0.44)
Change from baseline in rescue medication use, puffs per day, *n* ^a^			
Adjusted mean (SE)	−0.64 (0.24)	−0.68 (0.24)	−0.50 (0.23)
Difference in means versus placebo (95% CI)	..	−0.04 (−0.67 to 0.58)	0.14 (−0.48 to 0.77)
Asthma‐related healthcare use			
Number of events	18	7	6
Follow‐up time, years	98.4	100.8	105.1
Adjusted healthcare use rate, per year	0.21	0.08	0.06
Rate difference versus placebo, events/year	..	−0.13	−0.15
Rate ratio versus placebo (95% CI)	..	0.40 (0.16 to 1.00)	0.27 (0.10 to 0.72)

Abbreviations: ACQ‐5, Five‐Item Asthma Control Questionnaire; AQLQ +12, Asthma Quality of Life Questionnaire for 12 years and older; FeNO, fractional exhaled nitric oxide; FEV_1_, forced expiratory volume in 1 s; ITT, intention‐to‐treat; ppb, parts per billion.

^a^ Imputation of missing values was performed using the last observation carried forward

^b^ For missing responses, the domain score was calculated using the mean of those questions with an answer present, provided that ≥50% of the questions had an answer present; if <50% of the answers were present, the domain score was set to missing.

The proportions of patients who experienced treatment‐emergent adverse events (combined lebrikizumab doses, 155 [68%] of 229 patients vs. placebo, 72 [62%] of 117 patients), serious adverse events (seven [3%] patients vs. four [3%] patients), and adverse events leading to study drug discontinuation (five [2%] patients vs. one [1%] patient) were similar between lebrikizumab and placebo (Table 3 and appendix). No deaths occurred. Treatment groups were balanced on the key safety events of injection‐site reactions and serious infections, with no malignancies reported in any treatment group. Two cases evaluated as potential anaphylaxis, one case of tuberculosis, and one case of serious eosinophilia were reported (appendix). A 13‐year‐old male patient treated with lebrikizumab 37.5 mg developed itchy throat, facial swelling, and wheezing 3 days following the fourth dose of study drug; the patient was diagnosed with urticaria and severe angioedema, and the blinded investigator did not consider the event related to study drug. A 16‐year‐old female patient treated with lebrikizumab 125 mg experienced chest tightness and dyspnoea 4 days following the second dose of study drug; she went to the emergency department and was treated with epinephrine, methylprednisolone, and promethazine; the event resolved by the following day and was not considered related to study drug by the investigator. A 16‐year‐old female patient treated with lebrikizumab 125 mg presented with dyspnoea, cough, fever, and night sweats on study Day 101; cultures were positive for *Mycobacterium tuberculosis* sensitive to rifampicin; the patient was treated with oral ethambutol/isoniazid/pyrazinamide/rifampicin, and the event was recorded as “resolved” and not considered by the investigator to be related to study treatment. A 12‐year‐old female patient treated with lebrikizumab 37.5 mg developed serious eosinophilia (increase from 810 cells/μl at screening to 4770 cells/μl on study Day 85); no clinical signs or symptoms suggested an underlying pathology, and the blood eosinophil count decreased to 1110 cells/μl following discontinuation of study drug (study Day 107); the investigator considered the event related to study drug.

**TABLE 3:** Adverse events^a^

	Placebo (*n* = 117)	Lebrikizumab 37.5 mg (*n* = 113)	Lebrikizumab 125 mg (*n* = 116)	All lebrikizumab (*n* = 229)
Drug exposure^b^				
Number of doses, mean (SD)	9.7 (4.5)	10.1 (4.2)	10.4 (3.9)	10.2 (4.1)
Duration of treatment, mean (SD), weeks	39.1 (18.0)	40.5 (16.7)	41.9 (15.8)	41.2 (16.2)
Adverse events, *n*	300	247	279	526
Serious adverse events, *n*	4	4	5	9
Deaths, *n*	0	0	0	0
Patients with ≥1, *n* (%)				
Adverse event	72 (61.5%)	76 (67.3%)	79 (68.1%)	155 (67.7%)
Severe adverse event (at greatest intensity)	4 (3.4%)	6 (5.3%)	3 (2.6%)	9 (3.9%)
Adverse event assessed as related to study drug by investigator	9 (7.7%)	15 (13.3%)	16 (13.8%)	31 (13.5%)
Serious adverse event	4 (3.4%)	3 (2.7%)	4 (3.4%)	7 (3.1%)
Adverse event leading to discontinuation from study treatment	1 (0.9%)	3 (2.7%)	2 (1.7%)	5 (2.2%)
Adverse event of special interest				
Injection‐site reaction	6 (5.1%)	6 (5.3%)	11 (9.5%)	17 (7.4%)
Anaphylaxis per Sampson's criteria	0	1 (0.9%)	1 (0.9%)	2 (0.9%)
Assessed as related to study drug	0	1 (0.9%)	0	1 (0.4%)
Infection (broad definition)	53 (45.3%)	54 (47.8%)	54 (46.6%)	108 (47.2%)
Infection (narrow definition)^c^	0	0	1 (0.9%)	1 (0.4%)
Malignancy	0	0	0	0

MedDRA HLGT, Medical Dictionary for Regulatory Activities High‐Level Group Term.

^a^ Reflects total time in the study (up to 6 weeks following the last dose of drug).

^b^ Drug exposure during the 52‐week placebo‐controlled period.

^c^ MedDRA HLGT for helminthic disorders, mycobacterial infectious disorders, and protozoal infectious disorders or MedDRA HLGT of *Listeria* infections.

## 4 DISCUSSION

The results of this Phase 3 trial in adolescent patients with uncontrolled asthma indicate that treatment with lebrikizumab 125 mg every 4 weeks reduced asthma exacerbation rates. Exacerbation rates were reduced to a greater extent with both lebrikizumab dose levels (125 and 37.5 mg) in patients with higher (≥300 cells/μl) versus lower (<300 cells/μl) eosinophil counts. Analysis of secondary and exploratory endpoints demonstrated an increase in FEV_1_ with lebrikizumab 37.5 mg and an increase in time to first asthma exacerbation with both doses.

In patients with asthma, addressing uncontrolled disease despite adherence to standard‐of‐care treatments is a critical unmet need. Previous clinical trials in adult asthma have demonstrated that type 2 biomarkers, including blood and sputum eosinophils were correlated to severe airway inflammation, persistent symptoms, frequent exacerbations, and the clinical efficacy of these biomarkers in predicting treatment outcomes of type 2‐targeting biologics has been established.^20^ Furthermore, there are limited data on treatment of adolescent patients with uncontrolled asthma, although the high burden of disease in this patient population has been well documented.^3^, ^4^ Studies of other anti–IL‐5, anti–IL‐13, anti–IL‐4Rα/IL‐13, and anti‐thymic stromal lymphopoietin biologics (benralizumab, mepolizumab, reslizumab, tralokinumab, dupilumab, and tezepelumab) have included only a small number of adolescent patients, limiting interpretation of data in this population.^21^, ^22^, ^23^, ^24^, ^25^, ^26^, ^27^, ^28^, ^29^ Unlike these other studies of uncontrolled asthma which enrolled adolescents and adults together or enrolled younger children, ACOUSTICS was a dedicated all‐comers study of adolescent patients.

The overall reduction in asthma exacerbation rates in this study was better than those observed in the Phase 3 trials in adults with asthma treated with lebrikizumab: in the LAVOLTA studies, biomarker‐high patients (periostin ≥50 ng/ml, blood eosinophil count ≥300 cells/μl, or both) treated with lebrikizumab 125 and 37.5 mg achieved 30% and 51% reductions, respectively (LAVOLTA I), or a 26% reduction (LAVOLTA II; both doses).^19^ In ACOUSTICS, adolescent patients in the overall population treated with lebrikizumab 125 and 37.5 mg experienced 51% and 40% reductions in asthma exacerbation rates, respectively, compared with placebo. However, in the high eosinophil group, the ACOUSTICS adolescent patients experienced a 56%–58% reduction in asthma exacerbation rates in contrast to the 39% and 60% in the high eosinophil group in LAVOLTA I and the 32% and 43% reduction in LAVOLTA II.

Limitations to this study are associated with premature closing of enrolment and study drug dosing. The results could be influenced by multiple factors that may have reduced the power to detect any differences between treatment groups, including the pattern of early discontinuations (favoured patients in the placebo group) and the decision by the sponsor to close the study prematurely. As such, no statistical analyses beyond point estimates and 95% CIs were performed, limiting the ability to make meaningful comparisons between treatment groups. At the time of study design, which was prior to 2015, the understanding of biomarkers for asthma were unclear. Therefore, patients with exacerbations in the previous year were not included. Periostin was not measured because these were adolescent patients, and periostin is a bone growth marker which would be elevated and highly variable in adolescents and thus not a good biomarker in this age group. We speculate that 37.5 and 125 mg doses may not be high enough, as a dose response was seen in the atopic dermatitis trials which used much higher doses and increased frequency of dosing.^30^

In summary, this study found that treatment with lebrikizumab reduced the exacerbation rates in adolescent patients (greater effect observed with the 125‐mg dose than with the 37.5‐mg dose) and exacerbation rates were further reduced in patients who have baseline peripheral blood eosinophilia. A unique feature of this trial is that this is the largest free‐standing asthma study in adolescent patients. Current asthma trials include adolescents and adults into a single trial, with adolescent patients comprising a small subset of the overall adult population. Lebrikizumab showed a favourable safety profile in adolescents that was consistent with observations from adult studies.

## CONFLICT OF INTEREST

Stanley J. Szefler reports consultancy during the time of this report and currently for Aerocrine, AstraZeneca, Boehringer Ingelheim, Daiichi Sankyo, GlaxoSmithKline, Genentech, Inc., Merck, Novartis, Propeller Health, Regeneron, Roche, Sanofi and Teva and research support from the US National Institutes of Health; US National Heart, Lung, and Blood Institute; US National Institute of Allergy and Infectious Diseases; US National Institute of Environmental Health Sciences; US Environmental Protection Agency; Cancer, Cardiovascular and Pulmonary Disease Program; and GlaxoSmithKline. Graham Roberts was the lead investigator in the United Kingdom for this study. Adalberto S. Rubin reports fees from AstraZeneca, Boehringer Ingelheim, GSK, Novartis, Roche, Bristol, and Sanofi. Stefan Zielen reports grants and personal fees from bene‐Arzneimittel GmbH, grants from ALK Arzneimittel, personal fees from Novartis GmbH, Boehringer Ingelheim, Lofarma GmbH, IMS HEALTH GmbH and Co. OHG, GSK, Stallergen, Procter and Gamble, Allergopharma GmbH, AstraZeneca, Sanofi/Pasteur, and Aimmune outside the submitted work. Piotr Kuna reports personal fees from Astra, Boehringer Ingelheim, Berlin Chemie Menarini, GSK, Lekam, Novartis, Polpharma, Mylan, Orion, Teva, Adamed. Oral Alpan reports no disclosures that pertain to this submission. Cécile T J Holweg, and Wendy S. Putnam are former employees of Genentech, Inc. (a member of the Roche Group). Qiang Chen is an employee of Roche (China) Holding Ltd. Janusz Kaminski is a former employee of Roche Products Ltd. John G. Matthews is a former employee of Genentech, Inc. Judith Anzures‐Cabrera and Nikhil Kamath are employees of Roche Products Ltd.

## AUTHOR CONTRIBUTIONS

Stanley J. Szefler, Graham Roberts, Adalberto S. Rubin, Stefan Zielen, Piotr Kuna, Oral Alpan, Judith Anzures‐Cabrera, Qiang Chen, Cécile T. J. Holweg, Janusz Kaminski, Wendy S. Putnam, John G. Matthews, and Nikhil Kamath contributed to the conception and design of the study. Stanley J. Szefler, Adalberto S. Rubin, Stefan Zielen, Piotr Kuna, and Oral Alpan were involved in study enrolment and data collection. Judith Anzures‐Cabrera performed the statistical analysis. Stanley J. Szefler, Graham Roberts, Adalberto S. Rubin, Stefan Zielen, Piotr Kuna, Oral Alpan, Judith Anzures‐Cabrera, Qiang Chen, Cécile T J Holweg, Janusz Kaminski, Wendy S Putnam, John G Matthews, and Nikhil Kamath were involved in data analysis and interpretation, manuscript writing, and revision. All authors have read and approved the final version for submission.

## Supporting information

Supporting Information S1

## Data Availability

Qualified researchers may request access to individual patient‐level data through the clinical study data request platform (www.clinicalstudydatarequest.com). Further details on Roche's criteria for eligible studies are available here (https://clinicalstudydatarequest.com/Study‐Sponsors/Study‐Sponsors‐Roche.aspx). For further details on Roche's Global Policy on the Sharing of Clinical Information and how to request access to related clinical study documents, see here (https://www.roche.com/research_and_development/who_we_are_how_we_work/clinical_trials/our_commitment_to_data_sharing.htm).

## References

[1] Global Initiative for Asthma . GINA Report: Pocket Guide for Asthma Management and Prevention; 2020. Accessed 3 April 2020. https://ginasthma.org/wp‐content/uploads/2020/04/Main‐pocket‐guide_2020_04_03‐final‐wms.pdf

[2] Global Asthma Network . The Global Asthma Report 2018; 2018. Accessed 1 May 2019. http://globalasthmareport.org/

[3] Dolan CM , Fraher KE , Bleecker ER , et al. Design and baseline characteristics of the epidemiology and natural history of asthma: outcomes and Treatment Regimens (TENOR) study: a large cohort of patients with severe or difficult‐to‐treat asthma. Ann Allergy Asthma Immunol. 2004;92:32‐39. 10.1016/s1081-1206(10)61707-3 14756462

[4] Chipps BE , Szefler SJ , Simons FER , et al. Demographic and clinical characteristics of children and adolescents with severe or difficult‐to‐treat asthma. J Allergy Clin Immunol. 2007;119:1156‐1163. 10.1016/j.jaci.2006.12.668 17397912

[5] Chipps BE , Zeiger RS , Borish L , et al. Key findings and clinical implications from the epidemiology and natural history of asthma: outcomes and treatment regimens (TENOR) study. J Allergy Clin Immunol. 2012;130:332‐342. 10.1016/j.jaci.2012.04.014 22694932 PMC3622643

[6] National Asthma Education and Prevention Program . Expert panel report 3 (EPR‐3): guidelines for the diagnosis and management of asthma‐summary report 2007. J Allergy Clin Immunol. 2007;120(5 Suppl l):S94‐S138. 17983880 10.1016/j.jaci.2007.09.043

[7] Fajt ML , Wenzel SE . Asthma phenotypes and the use of biologic medications in asthma and allergic disease: the next steps toward personalized care. J Allergy Clin Immunol. 2015;135:299‐310. ; quiz 311. 10.1016/j.jaci.2014.12.1871 25662302

[8] Wenzel SE . Asthma phenotypes: the evolution from clinical to molecular approaches. Nat Med. 2012;18:716‐725. 10.1038/nm.2678 22561835

[9] Wenzel SE . Complex phenotypes in asthma: current definitions. Pulm Pharmacol Ther. 2013;26:710‐715. 10.1016/j.pupt.2013.07.003 23880027

[10] Moore WC , Bleecker ER , Curran‐Everett D , et al. Characterization of the severe asthma phenotype by the national Heart, lung, and blood institute's severe asthma research program. J Allergy Clin Immunol. 2007;119:405‐413. 10.1016/j.jaci.2006.11.639 17291857 PMC2837934

[11] Hershey GKK . IL‐13 receptors and signaling pathways: an evolving web. J Allergy Clin Immunol. 2003;111:677‐690; quiz 691. 10.1067/mai.2003.1333 12704343

[12] Grunig G , Warnock M , Wakil AE , et al. Requirement for IL‐13 independently of IL‐4 in experimental asthma. Science. 1998;282:2261‐2263. 10.1126/science.282.5397.2261 9856950 PMC3897229

[13] Humbert M , Durham SR , Kimmitt P , et al. Elevated expression of messenger ribonucleic acid encoding IL‐13 in the bronchial mucosa of atopic and nonatopic subjects with asthma. J Allergy Clin Immunol. 1997;99:657‐665. 10.1016/s0091-6749(97)70028-9 9155833

[14] Wills‐Karp M , Luyimbazi J , Xu X , et al. Interleukin‐13: central mediator of allergic asthma. Science. 1998;282:2258‐2261. 10.1126/science.282.5397.2258 9856949

[15] Corren J , Lemanske RF , Hanania NA , et al. Lebrikizumab treatment in adults with asthma. N Engl J Med. 2011;365:1088‐1098. 10.1056/nejmoa1106469 21812663

[16] Scheerens H , Arron JR , Zheng Y , et al. The effects of lebrikizumab in patients with mild asthma following whole lung allergen challenge. Clin Exp Allergy. 2014;44:38‐46. 10.1111/cea.12220 24131304 PMC4204278

[17] Ultsch M , Bevers J , Nakamura G , et al. Structural basis of signaling blockade by anti‐IL‐13 antibody Lebrikizumab. J Mol Biol. 2013;425:1330‐1339. 10.1016/j.jmb.2013.01.024 23357170

[18] Hanania NA , Noonan M , Corren J , et al. Lebrikizumab in moderate‐to‐severe asthma: pooled data from two randomised placebo‐controlled studies. Thorax. 2015;70:748‐756. 10.1136/thoraxjnl-2014-206719 26001563 PMC4515999

[19] Hanania NA , Korenblat P , Chapman KR , et al. Efficacy and safety of lebrikizumab in patients with uncontrolled asthma (LAVOLTA I and LAVOLTA II): replicate, phase 3, randomised, double‐blind, placebo‐controlled trials. Lancet Respir Med. 2016;4:781‐796. 10.1016/s2213-2600(16)30265-x 27616196

[20] Lee Y , Quoc QL , Park HS . Biomarkers for severe asthma: lessons from longitudinal cohort studies. Allergy Asthma Immunol Res. 2021;13(3):375‐389. 33733634 10.4168/aair.2021.13.3.375 PMC7984946

[21] Bleecker ER , FitzGerald JM , Chanez P , et al. Efficacy and safety of benralizumab for patients with severe asthma uncontrolled with high‐dosage inhaled corticosteroids and long‐acting beta2‐agonists (SIROCCO): a randomised, multicentre, placebo‐controlled phase 3 trial. Lancet. 2016;388:2115‐2127. 10.1016/s0140-6736(16)31324-1 27609408

[22] Ortega HG , Liu MC , Pavord ID , et al. Mepolizumab treatment in patients with severe eosinophilic asthma. N Engl J Med. 2014;371:1198‐1207. 10.1056/nejmoa1403290 25199059

[23] Pavord ID , Korn S , Howarth P , et al. Mepolizumab for severe eosinophilic asthma (DREAM): a multicentre, double‐blind, placebo‐controlled trial. Lancet. 2012;380:651‐659. 10.1016/s0140-6736(12)60988-x 22901886

[24] Bjermer L , Lemiere C , Maspero J , Weiss S , Zangrilli J , Germinaro M . Reslizumab for inadequately controlled asthma with elevated blood eosinophil levels: a randomized phase 3 study. Chest. 2016;150:789‐798. 10.1016/j.chest.2016.03.032 27056586

[25] Baverel PG , Jain M , Stelmach I , et al. Pharmacokinetics of tralokinumab in adolescents with asthma: implications for future dosing. Br J Clin Pharmacol. 2015;80:1337‐1349. 10.1111/bcp.12725 26182954 PMC4693499

[26] Castro M , Corren J , Pavord ID , et al. Dupilumab efficacy and safety in moderate‐to‐severe uncontrolled asthma. N Engl J Med. 2018;378:2486‐2496. 10.1056/nejmoa1804092 29782217

[27] Maspero JF , FitzGerald JM , Pavord ID , et al. Dupilumab efficacy in adolescents with uncontrolled, moderate‐to‐severe asthma: liberty asthma quest. Allergy. 2021;76:2621‐2624. 10.1111/all.14872 33905544 PMC8360078

[28] Menzies‐Gow A , Corren J , Bourdin A , et al. Tezepelumab in adults and adolescents with severe, uncontrolled asthma. N Engl J Med. 2021;384(19):1800‐1809. 33979488 10.1056/NEJMoa2034975

[29] Bacharier LB , Maspero JF , Katelaris CH , et al. Dupilumab in children with uncontrolled moderate‐to‐severe asthma. N Engl J Med. 2021;385(24):2230‐2240. 34879449 10.1056/NEJMoa2106567

[30] Silverberg JI , Thaçi D , Seneschal J , et al. Poster presented at: 4^th^ Annual Revolutionizing Atopic Dermatitis Conference; April 9‐11 2022. Accessed 8 June 2022. Efficacy and safety of lebrikizumab in moderate‐to‐severe atopic dermatitis: results from two phase 3, randomized, double‐blinded, placebo‐controlled trials. https://djbpnesxepydt.cloudfront.net/radv/April2022Posters/205‐ADvocate‐1‐‐‐2‐Primary‐16‐Wk_Silverberg‐et‐al_Poster_1649533037496.pdf

