# Supplementary material for: Efficacy, safety, and tolerability of lebrikizumab in adolescent patients with uncontrolled asthma (ACOUSTICS)
Source: Clin Transl Allergy. 2022 Jul 14;12(7):e12176. doi: 10.1002/clt2.12176 (PMC9281483; doi:10.1002/clt2.12176)
Supplement: Supplementary file 1 — Supporting Information S1 [file CLT2-12-e12176-s001.docx]

**Efficacy, safety, and tolerability of lebrikizumab in adolescent patients with uncontrolled asthma (ACOUSTICS)**

**Supplementary Appendix**

**Supplementary methods**

**Study protocol amendments**

Amendments to reduce patient and site burden related to the study assessments and enhance patient recruitment included conversion of the active-treatment extension to optional for patients; reduction in the frequency of certain assessments, including blood draws, spirometry, fractional exhaled nitric oxide (FeNO), urinalysis, and patient-reported outcomes (PROs); and elimination of the visit at Week 16 of the safety follow-up period.

**Recruitment and patient disposition**

Recruitment occurred from July 2013 to July 2016. Study dosing closed as of July 4, 2016, and the last patient’s last visit was on December 28, 2016.

**Randomisation and masking**

Patients were randomly assigned to the treatment groups through an interactive web-based response system (IxRS). Study-drug kit assignments were made by the IxRS service provider during the placebo-controlled period and the optional active-treatment extension. Patient randomisation and the study-drug kit assignments were verified on an ongoing basis by an external and independent statistical coordinating centre to ensure that randomisation and kit assignments were conducted correctly by the IxRS. The dynamic randomisation method is a generalisation of the hierarchical method proposed by Signorini and colleagues.^1^ A biased-coin assignment was used when the imbalance within a stratum exceeded a specified threshold.

Lebrikizumab and placebo were identical in appearance and were supplied by Roche in two configurations: a prefilled syringe and a vial. The 125-mg dose of lebrikizumab was supplied as a prefilled syringe, and the 37·5-mg dose was supplied in a vial. All patients received an injection from the prefilled syringe and from the vial to ensure masking of study treatment. Patients, investigators, study site personnel, and the funder were masked to treatment assignment during the study.

**Selection of lebrikizumab doses**

Given the lack of a clear dose-response relationship in Phase 2 studies, two dose levels, 125 and 37·5 mg, were chosen for the Phase 3 programme to conduct further dose ranging of lebrikizumab in the target population.^2^ The dosing frequency was supported by lebrikizumab’s half-life of 26 days and the safety and efficacy data from Phase 2 trials using the same dosing frequency.^2,3^ Based on data from Phase 2 studies, a dose of 125 mg was expected to demonstrate clinical efficacy in Phase 3 trials, whereas a lower dose of 37·5 mg (which corresponds to a 0·3-mL injection) was selected for dose ranging because it has a reasonable (3·3-fold) difference in dose from 125 mg, with minimum overlap in the simulated range of serum exposures between the two doses.

**Masking to pharmacodynamic biomarkers**

Patients and all study site personnel were masked to pharmacodynamic (PD) biomarker levels until study completion, except as described below. Sponsor study team members who had direct contact with study sites remained masked to individual FeNO levels, but team members who did not interact with study sites could review individual FeNO levels for assessment of baseline study information and ongoing review of data quality. The sponsor remained masked to individual posttreatment PD biomarker levels until completion of the placebo-controlled period. Eosinophil counts during screening were reviewed by the sites and the sponsor as part of routine laboratory data review for eligibility. Patients, all study site personnel, and the sponsor were masked to eosinophil counts after randomisation except for clinically significant elevations in eosinophil counts, which were reported to sites and could be reviewed by the sponsor as part of routine safety monitoring.

**Procedures**

Assessments included spirometry, measurement of biomarkers (FeNO, blood eosinophil count, total immunoglobulin E [IgE]), pharmacokinetics, antidrug antibodies, PRO measures (Five-Item Asthma Control Questionnaire [ACQ-5] and Asthma Quality of Life Questionnaire for 12 years and older [AQLQ +12]), and adverse events. Patients were provided with the e-Diary to record adherence to controller medication, their use of rescue medication, and peak expiratory flow (PEF).

The order of assessments conformed to the following requirements: asthma therapies that might affect spirometry were withheld until prebronchodilator spirometry measurements were completed. Patients were notified that use of twice-daily long-acting β-agonists (LABAs) and twice-daily long-acting muscarinic antagonists (LAMAs) was prohibited within 12 hours before spirometry, that use of once-daily LABAs and once-daily LAMAs was prohibited within 24 hours before spirometry, and that use of short-acting β-agonists or ipratropium was prohibited within 4 hours before spirometry. PRO questionnaires were administered before other assessments, and study drug was given after all other assessments had been completed.

Sites downloaded adherence data from the e-Diary at each visit and reviewed adherence to inhaled corticosteroids (ICS) and additional controller medications with the patient. Patient adherence to ICS and additional controller therapy, as recorded in the e-Diary, continued to be monitored throughout the placebo-controlled period. Patients also recorded measured PEF with the e-Diary. Once-daily PEF measurements consisted of three good efforts recorded between 5:00 am and 11:00 am daily before the morning inhaled medications were taken.

**Asthma exacerbations**

At each study visit, the investigator asked directed questions to assess whether the patient experienced any protocol-defined asthma exacerbations since the last study visit. An asthma exacerbation was defined as new or worsened asthma symptoms (including wheeze, cough, dyspnoea, chest tightness, and/or night time awakening due to these symptoms) that led to treatment with systemic corticosteroids or to hospitalisation. Treatment with systemic corticosteroids was defined as treatment with oral, intravenous, or intramuscular corticosteroids for ≥3 days or at least one dose of intravenous or intramuscular corticosteroids administered during an emergency department visit. Because asthma exacerbation rate was the primary endpoint in this study, a dedicated electronic case report form (eCRF) was used to record information regarding protocol-defined exacerbation events. An asthma exacerbation also had to be reported as an adverse event (or a serious adverse event, as applicable).

**Spirometry**

Spirometry, including the procedure for bronchodilator testing, was conducted at every visit on the basis of the American Thoracic Society (ATS)/European Respiratory Society (ERS) Consensus Statement "Standardisation of Spirometry."^2^ Measurement of spirometry was performed on a computerised spirometry system, Vitalograph Spirotrac, with 6800 Spirometer (Vitalograph; Ennis, Ireland) configured to the requirements of the study and in accordance with guidelines published by the ATS/ERS.^4^ For safety reasons, consideration was given to omitting spirometry if a patient was having an acute asthma exacerbation event at the time of the scheduled study visit. Spirometry measures included forced expiratory volume in 1 second (FEV_1_) and forced vital capacity (FVC; volume in L) and PEF (L/min). The percentage of predicted FEV_1_ and percentage of predicted FVC were derived from these volume measurements using the equations derived from the third US National Health and Nutrition Examination Survey.^5^ Asthma therapies that may affect spirometry were withheld until prebronchodilator spirometry measurements were completed. Patients were advised that inhaled bronchodilator use was prohibited within a specified window before each clinic visit, except for Visit 1, as follows: twice-daily LABA and twice-daily LAMA within 12 hours before spirometry, once-daily LABA and once-daily LAMA within 24 hours before spirometry, and short-acting β-agonist/ipratropium within 4 hours before the visit.

**Urgent asthma-related healthcare use**

At each study visit, the investigator asked directed questions to assess whether the patient had required any urgent asthma-related health care since the last study visit. Urgent asthma-related healthcare use included any hospitalisations, emergency department visits, and acute care visits (ie, unplanned clinic visits). Because urgent asthma-related healthcare use was a secondary efficacy endpoint in this study, a dedicated eCRF was used to record information regarding any asthma-related hospitalisations, emergency department visits, and/or acute care visits.

**Physician review of data captured using home electronic devices**

Electronic data (eg, daily PEF, rescue use, and asthma medication adherence) captured by the patient since the previous study visit were reviewed at each clinic visit. Data were reviewed to evaluate adherence to background medication and asthma status. Electronic data from the e-Diary were transferred to a web-based platform for review. Patients not adherent to their standard ICS and additional controller therapy from Visits 1 to 3, as evidenced by <70% adherence during the screening period, were not eligible for the study. However, the screening period could be extended by 1 week for additional adherence monitoring in the case of device malfunction or extenuating patient circumstances. When this occurred, the adherence calculation was based on the number of days the patient was adherent to baseline therapy when the device was used and functioning properly. Patient adherence to ICS and additional controller therapy, as recorded in the e-Diary, continued to be monitored throughout the placebo-controlled period.

**Patient-reported outcomes**

PRO (ACQ-5, AQLQ +12) data were collected and instruments were translated as required in the local language. The ACQ-5 and AQLQ +12 were completed in their entirety by the patient. To ensure instrument validity and to ensure that data standards met health authority requirements, the ACQ-5 and AQLQ +12 were self-administered at the investigational site prior to the completion of other non-PRO assessments and before the patient received any disease status information or study drug during that visit. PRO data were collected electronically using electronic PRO tablet devices.

*Five-Item Asthma Control Questionnaire*

Asthma control, as measured by the ACQ-5,^6-7^ was assessed at Weeks 0, 24, and 52 by asking patients to recall their experiences during the previous week and to respond to five questions (ie, night time awakening, symptoms on waking, activity limitation, shortness of breath, and wheeze). The ACQ-5 has a recall period of 1 week. The items were scored on a scale between 0 (good control) and 6 (poor control). The items were equally weighted, and the score was the mean of the five items. The ACQ-5 has been validated and has strong measurement properties for use in both clinical practice and clinical trials.

*Asthma Quality of Life Questionnaire* *for 12 years and older*

The AQLQ +12 was used to assess asthma-specific health-related quality of life.^8^ The 32-item questionnaire contains four domains: activity limitations, symptoms, emotional function, and environmental stimuli. The AQLQ +12 is based on the standardised version of the AQLQ, with a minor adaption to make it valid for use with asthmatic patients aged ≥12 years. The items on the AQLQ +12 were scored on a scale between 1 (severe impairment) and 7 (no impairment). The AQLQ +12 has a recall specification of 2 weeks.

**Fractional exhaled nitric oxide**

Measurement of FeNO was performed at every visit using a handheld portable NIOX MINO device (Aerocrine, Solna, Sweden) in accordance with guidelines published by the ATS. FeNO was performed before spirometry testing.

**Blood eosinophil count**

Blood samples were taken at every visit. Blood eosinophils were part of the whole blood count and were measured centrally on automated haematology analysers.

**Total immunoglobulin E**

Blood samples were taken throughout the study. Total IgE was measured using the ImmunoCAP test (Phadia AB, Uppsala, Sweden; assay performed by Viracor IBT (currently Viracor Eurofins Clinical Diagnostics, Dr. Lee’s Summit, MO, USA).

**C-C motif chemokine (CCL) - 13**

Blood samples were taken throughout the study. Serum C-C motif chemokine 13 (CCL13) was measured at Bio-Techne (Minneapolis, MN, USA) according to the company’s standard operating procedures.

**Antibodies**

Antibodies against lebrikizumab and putative phospholipase B-like 2 were detected using two validated bridging immunoassays, respectively. Samples that screened positive were further confirmed by immunodepletion.

**Power and sample size calculation**

ACOUSTICS planned to enrol a total of 375 patients to provide approximately 80% power to detect a 50% reduction in the asthma exacerbation rate with a given lebrikizumab dose level compared with placebo. Calculations were based on a Poisson regression model and 2-sided test at the α=0·05 level, with the assumptions of an average rate of 0·6 exacerbations per patient in the placebo group over a 52-week period, a 20% dropout rate by Week 52, and 20% Poisson overdispersion. Statistical power was evaluated using Monte Carlo simulations.

***Supplementary Figure S1:* Adjusted mean change in FEV_1_ over time**


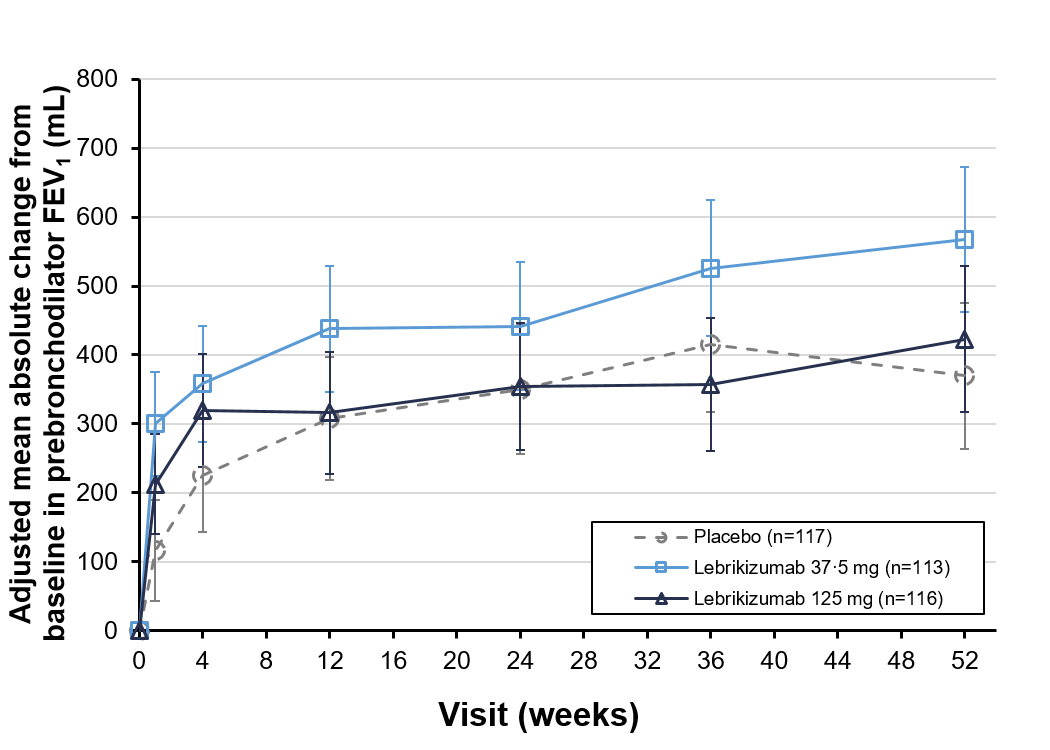


Bars represent 95% CIs. Estimates were based on a mixed-model repeated-measures analysis with an unstructured covariance matrix. The model used absolute change from baseline in prebronchodilator FEV_1_ as the response variable and included terms for treatment, visit, treatment × visit, baseline FEV_1_, baseline FEV_1_ × visit, number of asthma exacerbations within the last 12 months, baseline asthma medications, and age group. FEV_1_=forced expiratory volume in 1 second.

***Supplementary Figure S2:* Change from baseline in serum CCL13, serum IgE, and blood eosinophil count throughout the 52-week placebo-controlled period**

***
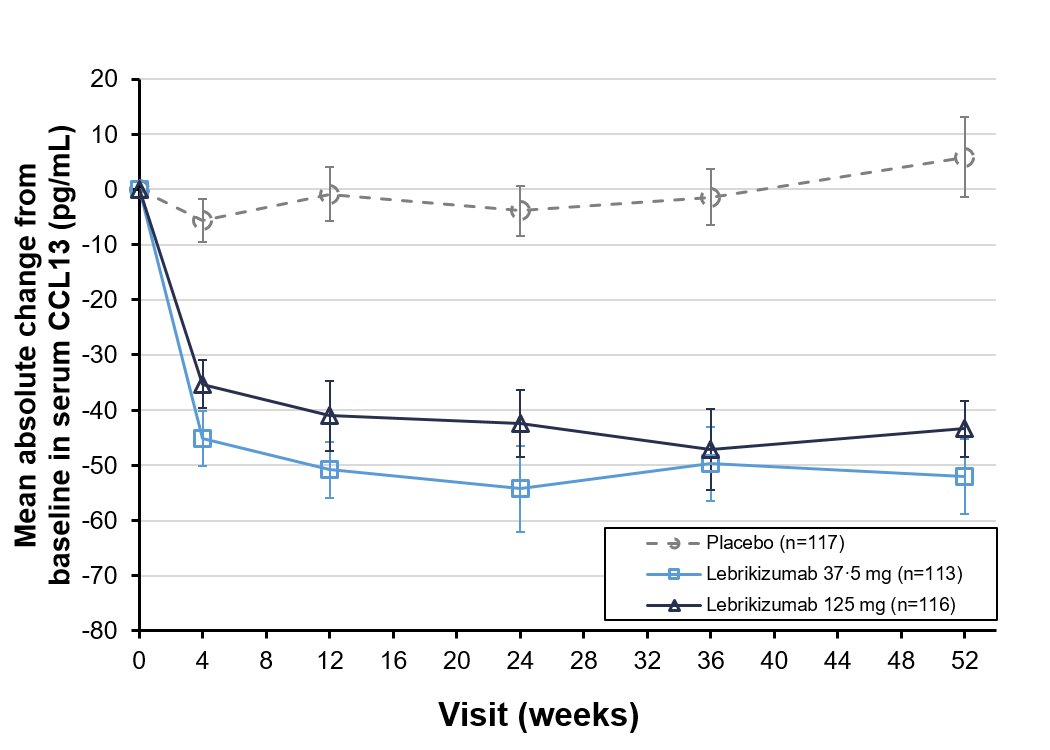
***

***
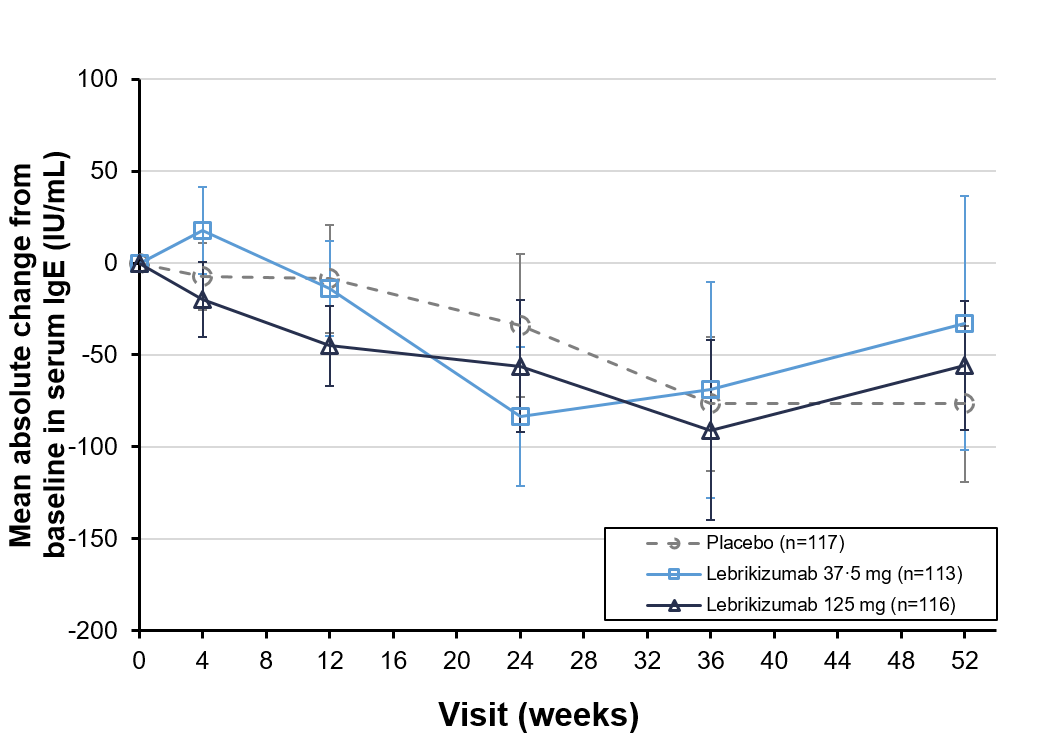
***

***
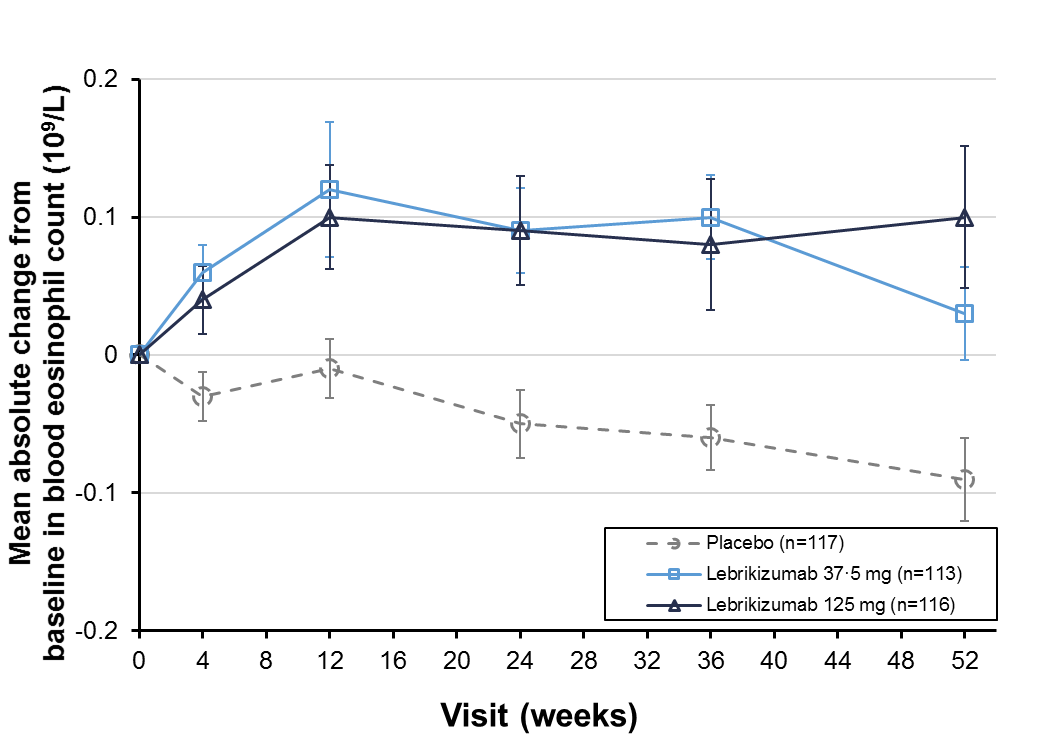
***

Error bars represent standard errors of the mean. CCL13=chemokine (C-C motif) ligand 13. IgE=immunoglobulin E.

Mean serum CCL13 and total IgE decreased after the administration of lebrikizumab and remained low throughout the placebo-controlled period, while blood eosinophil counts increased and remained elevated throughout the treatment period.

***Supplementary Table S1:* Exploratory efficacy estimates of change in ACQ‑5 score from baseline at 52 weeks (ITT population)**

|  | **Placebo**  **(n=117)** | **Lebrikizumab  37·5 mg (n=113)** | **Lebrikizumab  125 mg (n=116)** |
| --- | --- | --- | --- |
| Change from baseline in ACQ-5 score* |  |  |  |
| Adjusted mean (SE) | −1·04 (0·11) | −1·38 (0·11) | −1·34 (0·11) |
| Difference in means *vs* placebo (95% CI) | ·· | −0·34 (−0·63 to −0·05) | −0·30 (−0·59 to 0·00) |

ACQ-5=Five-Item Asthma Control Questionnaire. *Imputation of missing values was performed using the last observation carried forward.

An exploratory analysis examining change in ACQ-5 scores from baseline to Week 52 showed that differences from placebo did not reach the threshold of minimal clinically important difference (0·5) with either lebrikizumab dose.

***Supplementary Table S2:* Secondary efficacy outcomes at 52 weeks by eosinophil subgroup**

|  | **Eosinophil high (≥300 cells/µL)** | | | **Eosinophil low (<300 cells/µL)** | | |
| --- | --- | --- | --- | --- | --- | --- |
|  | **Placebo**  **(n=65)** | **Lebrikizumab  37·5 mg (n=50)** | **Lebrikizumab  125 mg (n=58)** | **Placebo**  **(n=52)** | **Lebrikizumab  37·5 mg (n=63)** | **Lebrikizumab  125 mg (n=58)** |
| Change from baseline in prebronchodilator FEV_1_, mL |  |  |  |  |  |  |
| Adjusted mean (SE) | 309 (72) | 534 (78) | 435 (75) | 439 (81) | 602 (74) | 415 (78) |
| Difference in means (95% CI) | ·· | 225 (21 to 429) | 126 (−73 to 325) | ·· | 163 (−46 to 373) | −24 (−240 to 193) |
| Time to first exacerbation |  |  |  |  |  |  |
| Patients with event, n (%) | 21 (32%) | 8 (16%) | 12 (21%) | 12 (23%) | 10 (16%) | 8 (14%) |
| Hazard ratio (95% CI) | ·· | 0·35 (0·15 to 0·86) | 0·42 (0·19 to 0·90) | ·· | 0·51 (0·20 to 1·31) | 0·39 (0·15 to 1·01) |
| Change from baseline in prebronchodilator FeNO, ppb |  |  |  |  |  |  |
| Adjusted mean (SE) | 2 (4.5) | −26 (5) | −39 (4.5) | 4 (3) | −9 (2) | −13 (3) |
| Difference in means (95% CI) | ·· | −28 (−41 to −15) | −41 (−53 to −29) | ·· | −13 (−20 to −7) | −17 (−24 to −10) |
| Change from baseline in ACQ-5 score |  |  |  |  |  |  |
| Adjusted mean (SE) | −1·10 (0·16) | −1·44 (0·16) | −1·47 (0·16) | −1·06 (0·17) | −1·30 (0·16) | −1·11 (0·17) |
| Difference in means (95% CI) | ·· | −0·34 (−0·75 to 0·06) | −0·37 (−0·77 to 0·03) | ·· | −0·24 (−0·67 to 0·19) | −0·05 (−0·50 to 0·40) |

FEV_1_=forced expiratory volume in 1 second. FeNO=fractional exhaled nitric oxide. ppb=parts per billion. ACQ-5=Five-Item Asthma Control Questionnaire.

***Supplementary Table S3:* Pharmacokinetics: trough concentrations of lebrikizumab over 52-week placebo-control period**

| **Treatment group, mean (SD; n)** | **C_min_, Week 4, µg/mL** | **C_min_, Week 12, µg/mL** | **C_min_, Week 24, µg/mL** | **C_min_, Week 36, µg/mL** | **C_min_, Week 52, µg/mL** |
| --- | --- | --- | --- | --- | --- |
| Lebrikizumab 37·5 mg | 3·25 (1·58; 47) | 5·72 (2·81; 42) | 7·15 (6·23; 28) | 3.88 (2.92; 18) | NR (4) |
| Age 12–14 years | 3·50 (2·02; 24) | 5·96 (2·82; 22) | 7·88 (7·23; 15) | 3·43 (2·47; 9) | NR (3) |
| Age 15–17 years | 3·00 (0·92; 23) | 5·46 (2·85; 20) | 6·31 (5·00; 13) | 4·33 (3·40; 9) | NR (1) |
| Lebrikizumab 125 mg | 12·2 (5·28; 50) | 21·7 (9·37; 40) | 25·7 (9·84; 29) | 23·9 (9·87; 23) | NA |
| Age 12–14 years | 13·0 (5·31; 34) | 23·0 (8·77; 27) | 28·7 (7·37; 17) | 27·4 (8·29; 15) | NA |
| Age 15–17 years | 10·5 (4·95; 16) | 18·9 (10·3; 13) | 21·4 (11·6; 12) | 17·5 (9·79; 8) | NA |

C_min_=predose serum concentration. NR=non-reportable. NA=not available (no values reported at this time point).

The observed mean (SD) trough concentrations of study drug at the 125- and 37·5-mg dose levels were approximately dose proportional and approached steady state by Week 12. Trough concentrations were similar between the age groups of 12–14 and 15–17 years.

***Supplementary Table S4:* Prevalence and incidence of antidrug antibodies**

| **Positive test result for antitherapeutic antibodies, n positive (%; n available)*** | **Placebo**  **(n = 56)** | **All lebrikizumab (n = 290)** |
| --- | --- | --- |
| Patients with positive antidrug antibody test result at baseline visit | 1 (1·9%; 53) | 1 (0·4%; 262) |
| Patients with ≥1 positive antidrug antibody test result at any postbaseline visit | 0 (42) | 5 (1·9%; 260) |

*Samples for antidrug antibody analyses were obtained at baseline and at the last visit (including safety follow-up).

***Supplementary Table S5:* Serious adverse events**

| **n (%)** | **Placebo**  **(n=117)** | **Lebrikizumab  37·5 mg (n=113)** | **Lebrikizumab  125 mg (n=116)** | **All lebrikizumab (n=229)** |
| --- | --- | --- | --- | --- |
| Patients with ≥1 adverse event | 4 (3%) | 3 (3%) | 4 (3%) | 7 (3%) |
| Total events, n | 4 | 4 | 5 | 9 |
| Blood and lymphatic system disorders |  |  |  |  |
| Eosinophilia | 0 | 1 (1%) | 0 | 1 (<1%) |
| Gastrointestinal disorders |  |  |  |  |
| Diarrhoea | 1 (1%) | 0 | 0 | 0 |
| Immune system disorders |  |  |  |  |
| Anaphylactic reaction | 0 | 0 | 1 (1%) | 1 (<1%) |
| Infections and infestations |  |  |  |  |
| Pulmonary tuberculosis | 0 | 0 | 1 (1%) | 1 (<1%) |
| Sinusitis | 1 (1%) | 0 | 0 | 0 |
| Metabolism and nutrition disorders |  |  |  |  |
| Metabolic syndrome | 0 | 0 | 1 (1%) | 1 (<1%) |
| Nervous system disorders |  |  |  |  |
| Dizziness | 0 | 0 | 1 (1%) | 1 (<1%) |
| Respiratory, thoracic, and mediastinal disorders |  |  |  |  |
| Asthma | 2 (2%) | 0 | 1 (1%) | 1 (<1%) |
| Asthmatic crisis | 0 | 1 (1%) | 0 | 1 (<1%) |
| Skin and subcutaneous tissue disorders |  |  |  |  |
| Angioedema | 0 | 1 (1%) | 0 | 1 (<1%) |
| Urticaria | 0 | 1 (1%) | 0 | 1 (<1%) |

Data presented by Medical Dictionary for Regulatory Activities system organ class (heading) and preferred term (subheading).

***Supplementary Table S6:* Adverse events of special interest—infections (broad)**

| **n (%)** | **Placebo**  **(n=117)** | **Lebrikizumab 37·5 mg (n=113)** | **Lebrikizumab 125 mg (n=116)** | **All lebrikizumab (n=229)** |
| --- | --- | --- | --- | --- |
| Patients with ≥1 adverse event | 53 (45%) | 54 (48%) | 54 (47%) | 108 (47%) |
| Total events, n | 113 | 93 | 93 | 186 |
| Infections and infestations |  |  |  |  |
| Nasopharyngitis | 12 (10%) | 13 (12%) | 12 (10%) | 25 (11%) |
| Pharyngitis | 13 (11%) | 6 (5%) | 15 (13%) | 21 (9%) |
| Upper respiratory tract infection | 7 (6%) | 13 (12%) | 7 (6%) | 20 (9%) |
| Bronchitis | 8 (7%) | 6 (5%) | 6 (5%) | 12 (5%) |
| Tonsillitis | 6 (5%) | 7 (6%) | 7 (6%) | 14 (6%) |
| Sinusitis | 6 (5%) | 2 (2%) | 3 (3%) | 5 (2%) |
| Rhinitis | 6 (5%) | 3 (3%) | 1 (1%) | 4 (2%) |
| Influenza | 2 (2%) | 2 (2%) | 5 (4%) | 7 (3%) |
| Acute sinusitis | 4 (3%) | 1 (1%) | 2 (2%) | 3 (1%) |
| Viral upper respiratory tract infection | 4 (3%) | 1 (1%) | 2 (2%) | 3 (1%) |
| Gastroenteritis | 3 (3%) | 1 (1%) | 2 (2%) | 3 (1%) |
| Respiratory tract infection | 3 (3%) | 1 (1%) | 2 (2%) | 3 (1%) |
| Conjunctivitis | 2 (2%) | 1 (1%) | 0 | 1 (<1%) |
| Laryngitis | 1 (1%) | 1 (1%) | 1 (1%) | 2 (1%) |
| Otitis media acute | 1 (1%) | 2 (2%) | 0 | 2 (1%) |
| Pneumonia | 1 (1%) | 0 | 2 (2%) | 2 (1%) |
| Viral infection | 0 | 1 (1%) | 2 (2%) | 3 (1%) |
| Ear infection | 0 | 2 (2%) | 0 | 2 (1%) |
| Epididymitis | 1 (1%) | 0 | 1 (1%) | 1 (<1%) |
| Gastroenteritis viral | 0 | 1 (1%) | 1 (1%) | 2 (1%) |
| Otitis media | 1 (1%) | 0 | 1 (1%) | 1 (<1%) |
| Tracheobronchitis | 1 (1%) | 0 | 1 (1%) | 1 (<1%) |
| Urinary tract infection | 1 (1%) | 1 (1%) | 0 | 1 (<1%) |
| Cellulitis | 0 | 1 (1%) | 0 | 1 (<1%) |
| Chikungunya virus infection | 0 | 0 | 1 (1%) | 1 (<1%) |
| Conjunctivitis viral | 0 | 1 (1%) | 0 | 1 (<1%) |

Data are n (%) and are presented by Medical Dictionary for Regulatory Activities system organ class (heading) and preferred term (subheading).

***Supplementary Table S7:* Adverse events of special interest—infections (narrow)**

| **Treatment group** | **Event term** | **Clinical history** |
| --- | --- | --- |
| 125 mg | Tuberculosis | A 16-year-old female patient presented with dyspnoea, cough, fever, and night sweats on study Day 101. The sputum culture was positive for *Mycobacterium tuberculosis* sensitive to rifampicin, and a polymerase chain reaction test also yielded a positive result. The patient was treated with ethambutol/isoniazid/pyrazinamide/rifampicin, and the event was recorded as “resolved.” |

***Supplementary Table S8:* Anaphylaxis events**

| **Treatment group** | **Event term** | **Clinical history** |
| --- | --- | --- |
| 37·5 mg | Anaphylaxis | A 13-year-old male patient, 3 days following the fourth dose of study drug, developed itchy throat, facial swelling, and wheeze. The patient received intramuscular epinephrine. C3 and C4 complement proteins and serum tryptase levels were reported as normal. An allergist was consulted and did not consider the event to be true anaphylaxis. The patient was diagnosed with urticaria and severe angioedema. The principal investigator did not consider the event related to study drug. |
| 125 mg | Anaphylaxis | A 16-year-old female patient, 4 days following the second dose of study drug, experienced chest tightness and dyspnoea and was treated with asthma medication. The same evening, the patient ate “pickles” and developed angioedema, itchy throat, hives, and pruritus. The patient attended the emergency department and was treated with epinephrine, methylprednisolone, and promethazine. The event resolved by the following day. |

***Supplementary Table S9:* Eosinophil-related adverse events**

| **Treatment group** | **Event term** | **Clinical history** |
| --- | --- | --- |
| 37·5 mg | Eosinophilia (serious) | A 12-year-old female patient had a blood eosinophil count of 810 cells/µL at screening. On study Day 85 (after 5 doses of study drug), the blood eosinophil count increased to 4770 cells/µL. No clinical signs or symptoms were reported at this time to suggest any underlying pathology; electrocardiography, echocardiogram, and bronchial fibroscopy results were all normal. Study drug was discontinued. Following discontinuation of the study drug, her blood eosinophil count decreased to 1110 cells/µL (study Day 107). |
| 125 mg | Eosinophilia  (non-serious) | A 12-year-old female patient had a blood eosinophil count of 870 cells/µL at screening. An event of eosinophilia was reported on study Day 208. On study Day 237, parasite screening results were positive for *Endolimax nana*; the patient was diagnosed with a mild parasitic infection (non-serious, unrelated). No treatment was given for the eosinophilia. The principal investigator considered the event of eosinophilia as possibly related to parasitic infection. |

***Supplementary Table S10:* Principal investigators and sites**

| **Country** | **Investigator** | **City*** |
| --- | --- | --- |
| Argentina | H Altieri | Tucumán |
| Argentina | M Bergna | Vicente López |
| Argentina | C Fazio | Mendoza |
| Argentina | M Maillo | Santa Fe |
| Argentina | A Stok | San Miguel De Tucumán |
| Argentina | A Yañez | Buenos Aires |
| Brazil | M Antila | São Paulo |
| Brazil | J Fiterman | Rio Grande do Sul |
| Brazil | C Galvão | São Paulo |
| Brazil | M Neis | São Paulo |
| Brazil | A Pez | Santo André |
| Brazil | A Rubin | Rio Grande do Sul |
| Canada | B Lyttle | London, ON |
| Colombia | J Aristizabal Duque | Bogotá |
| Czech Republic | T Hofstetr | Jihlava |
| Czech Republic | J Vankova | Teplice |
| France | C Marguet | Rouen |
| Germany | W Kamin | Hamm |
| Germany | S Zielen | Frankfurt am Main |
| Hungary | É Gács | Budapest |
| Hungary | G Mezei | Budapest |
| Hungary | Á Németh | Budapest |
| Hungary | G Papp | Szigetvár |
| Hungary | I Varkonyi | Debrecen |
| Israel | L Bentur-Alkobi | Haifa |
| Israel | O Efrati | Ramat-Gan |
| Israel | M Mei Zahav | Petach Tikva |
| Israel | E Picard | Jerusalem |
| Italy | G L Marseglia | Pavia |
| Mexico | R Cerino Javier | Villahermosa |
| Mexico | D Hernandez Colin | Guadalajara |
| Mexico | E Montano Gonzalez | Guadalajara |
| Mexico | A Ramirez | Monterrey |
| Mexico | G Velazquez | Benito Juarez |
| Peru | A Guerreros Benavides | Lima |
| Poland | M Bederska | Krarkow |
| Poland | A Brzozowska | Łódź^†^ |
| Poland | A Fal | Warszawa |
| Poland | T Hofman | Poznan |
| Poland | P Kuna | Łódź |
| Poland | B Majorek-Olechowska | Tarnów |
| Portugal | A Morete | Aveiro |
| Portugal | C Nunes | Faro |
| Slovakia | M Dzurilla | Nitra |
| South Africa | Q Bhorat | Johannesburg |
| South Africa | I Mitha | Benoni |
| South Africa | S Mogashoa | Pretoria |
| South Africa | P Sebastian | Durban |
| South Africa | R van Zyl-Smit | Cape Town |
| Spain | M P Alba Jorda | Valencia^‡^ |
| Spain | O Asensio de la Cruz | Cataluña |
| Spain | R J Calderón Fernandez | Valencia^‡^ |
| Spain | A Roger | Barcelona |
| Ukraine | L Bezrukov | Chernivtsi |
| Ukraine | K Duka | Dnipro |
| Ukraine | V Kostromina | Kyiv |
| Ukraine | V Lapshyn | Kyiv |
| Ukraine | S Mokiya-Serbina | Kryvyi Rih |
| Ukraine | Y Reznychenko | Zaporizhzhya |
| Ukraine | I Stepanova | Kyiv |
| United Kingdom | B Neupane | Leicester^‡^ |
| United Kingdom | T Ninan | Birmingham |
| United Kingdom | H Pandya | Leicester^‡^ |
| United States | O Alpan | Fairfax, VA |
| United States | J Bennion | North Logan, UT |
| United States | G Bensch | Stockton, CA |
| United States | W Berger | Mission Viejo, CA |
| United States | T Bridges | Albany, GA |
| United States | S DeLeon | Bronx, NY |
| United States | J Diaz | San Antonio, TX |
| United States | D Fuentes | Boerne, TX |
| United States | J Gomez | Miami, FL |
| United States | E Gonzalez-Reyes | San Antonio, TX |
| United States | I Melamed | Centennial, CO |
| United States | R Nathan | Colorado Springs, CO |
| United States | S Sekhsaria | White Marsh, MD |
| United States | S Szefler | Aurora, CO |
| United States | H Windom | Sarasota, FL |
| United States | B Yu | Chicago, IL |

* Cities with ≥1 investigator and/or study site are listed once for each study site.

^†^ Study site underwent change of address during study (listed only once).

^‡^ Study site underwent change of principal investigator during study (both investigators listed).

**Supplementary References**

1. Signorini DF, Leung O, Simes RJ, Beller E, Gebski VJ, Callaghan T. Dynamic balanced randomization for clinical trials. *Stat Med* 1993; **12:** 2343–50.
2. Hanania NA, Noonan M, Corren J, et al. Lebrikizumab in moderate-to-severe asthma: pooled data from two randomised placebo-controlled studies. *Thorax* 2015; **70:** 748–56
3. Zhu R, Zheng Y, Dirks NL, et al. Model-based clinical pharmacology profiling and exposure-response relationships of the efficacy and biomarker of lebrikizumab in patients with moderate-to-severe asthma. *Pulm Pharmacol Ther* 2017; **46:** 88–98.
4. Miller MR, Hankinson J, Brusasco V, et al. Standardisation of spirometry. *Eur Respir J* 2005; **26:** 319–38.
5. Hankinson JL, Odencrantz JR, Fedan KB. Spirometric reference values from a sample of the general U.S. population. *Am J Respir Crit Care Med* 1999; **159:** 179–87.
6. Juniper EF, O'Byrne PM, Ferrie PJ, King DR, Roberts JN. Measuring asthma control. Clinic questionnaire or daily diary? *Am J Respir Crit Care Med* 2000; **162:** 1330–4.
7. Juniper EF, Svensson K, Mörk AC, Ståhl E. Measurement properties and interpretation of three shortened versions of the asthma control questionnaire. *Respir Med* 2005; **99:** 553–8.
8. Juniper EF, Svensson K, Mörk AC, Ståhl E. Modification of the asthma quality of life questionnaire (standardised) for patients 12 years and older. *Health Qual Life Outcomes* 2005; **3:** 58.
